# Ten years after an outbreak of *Fusarium* endophthalmitis following cataract surgery

**DOI:** 10.5935/0004-2749.20200106

**Published:** 2024-02-11

**Authors:** Neşe Alagöz

**Affiliations:** 1 Beyoğlu Eye Training and Research Hospital, University of Health Sciences, Istanbul, Turkey

## INTRODUCTION

*Fusarium* species are the commonly encountered agents in cases of fungal keratitis, that develop mostly secondary to trauma or contact lens use^(1)^. Although rare, *Fusarium* species have been reported to cause endophthalmitis after intraocular surgery^(2)^. The eradication of the fungus is extremely difficult in cases with in traocular involvement, and it requires aggressive treatment with multiple antifungal agents delivered topically, systemically, and intravitreally as well as multiple and repeated surgeries involving keratoplasty, pars plana vitrectomy, lensectomy/intraocular lens removal, and iridectomy. Thus, the final visual outcomes are not typically favorable in patients with fungal endophthalmitis, with up to 60% of eyes eventually being enucleated^(1,3)^.

Improvement in treatment modalities and the early identification of the microorganism has resulted in better management during the acute infectious phase and fewer eyes proceeding to evisceration as compared with the past. However, after eyes are salvaged, they require further management for visual rehabilitation. Herein, we present the long-term follow-up results of patients involved in a *Fusarium* endophthalmitis outbreak that occurred in 2008, report the additional surgical procedures that were applied for visual rehabilitation after the acute infectious phase subsided, and discuss the factors influencing the final long-term results.

## METHODS

We retrospectively reviewed the medical records of eight patients (three males, five females) who were involved in the fungal endophthalmitis outbreak after cataract surgery performed in the same operating room and on the same date. Although there was variation in surgeons, the common event for all patients was the administration of infused intraocular cefuroxime-axetil from the same vial for all cases. All specimens collected from the patients grew filamentous fungi of *Fusarium* species.

The study adhered to the tenets of the Declaration of Helsinki, and the local ethical committee approved the study.

## RESULTS

Early results regarding the acute-phase management and clinical outcomes were previously reported^(2)^. Table 1 summarizes the early and late results. In total, the infection was eradicated in seven eyes, whereas one eye underwent evisceration after corneal involvement and uncontrolled infection (Case 1).

**Table 1:** Patient characteristics, surgical interventions, and clinical results

Case	Age, years	Sex	Eye	Associated conditions	Presenting VA	Acute-phase surgical intervention (times)	VA(a)	Late-phase surgical intervention (times)	VA(I)	Status at last examination
1	49	M	R	DM	20/400	1V-AF (2), PPV (2), evisceration	-	-	-	Eviscerated
2	62	F	L	DM, PDR	HM	1V-AF (10), PPV (2)	LP(+)		LP(-)	Phthisic
3	70	M	R	Glaucoma, pterygium	1 mfc	1V-AF (2), PPV (4)	20 cmfc	CPC (3), Limbal stem cell transplantation (1), KP (1)	HM	Opacified and vascularized corneal graft, limbal stem cell deficiency, partial retinal detachment
4	76	F	L	Glaucoma, HT	20/200	1V-AF (3), PPV (2)	20/100	SiO removal (1), CPC (4), KP + 1OL implantation (1)	HM	Opacified and vascularized corneal graft, attached retina
5	64	F	L	Glaucoma	20/63	1V-AF (2), PPV(1)	20/63	SiO removal (1), KP (1)	20/400	Hazy corneal graft, aphakia, pale optic disc
6	74	M	L	-	20/25	1V-AF (1), PPV(1)	20/100	SiO removal + 1OL implantation (1)	20/80	Clear cornea, 1F-1OL, pale optic disc
7	78	F	R	Corneal dystrophy	50 cmfc	1V-AF (2), PPV(1)	30 cmfc	SiO removal (1)	30 cmfc	Hazy cornea, aphakia, pale optic disc (at 18 months) Patient died at 2 years
8	77	F	R		HM	1V-AF (3), PPV(1)	3 mfc	NA (lost to follow-up)	N/A	N/A

VA= visual acuity; VA(a)= visual acuity at the end of the acute phase; VA(l)= visual acuity at last follow-up, M= male; F= female, R= right eye; L= left eye; DM= diabetes mellitus= PDR= proliferative diabetic retinopathy; HT= systemic hypertension; HM= hand motion; mfc= meter finger count, cmfc= centimeter finger count; LP= light perception; IV-AF= intravitreal antifungal; PPV= pars plana vitrectomy; IOL= intraocular lens; IF-IOL= iris-fixated intraocular lens, KP= keratoplasty, SiO= silicon oil; CPC= cyclophotocoagulation;

N/A= not available.

## DISCUSSION

Concomitant systemic and ocular diseases seem to be important for the final functional outcome of patients with fungal endophthalmitis. Tissue injury resulting from the acute phase of the disease also appears to influence the long-term outcome.

All patients in our series, except for Case 1, were older than 60 years, and the mean age of the group was 69 years. A previous report defined advanced age as a risk factor for corneal involvement and poor prognosis in *Fusarium* endophthalmitis^(4)^. The risk factors for Case 1 (eviscerated in acute period) were corneal involvement in the course of the disease and systemic diabetes. Corneal involvement was reported to be the single most important independent predictor of poor final visual outcome in fungal endophthalmitis^(5)^. Case 2, who had unregulated diabetes and diabetic retinopathy, progressed to phthisis during the follow-up period. In our series, the two patients with surgically managed glaucoma developed graft failure and had a poor final visual outcome, thus pointing out the negative effect of glaucoma on graft survival (Cases 3 and 4).

**Figure 1:**
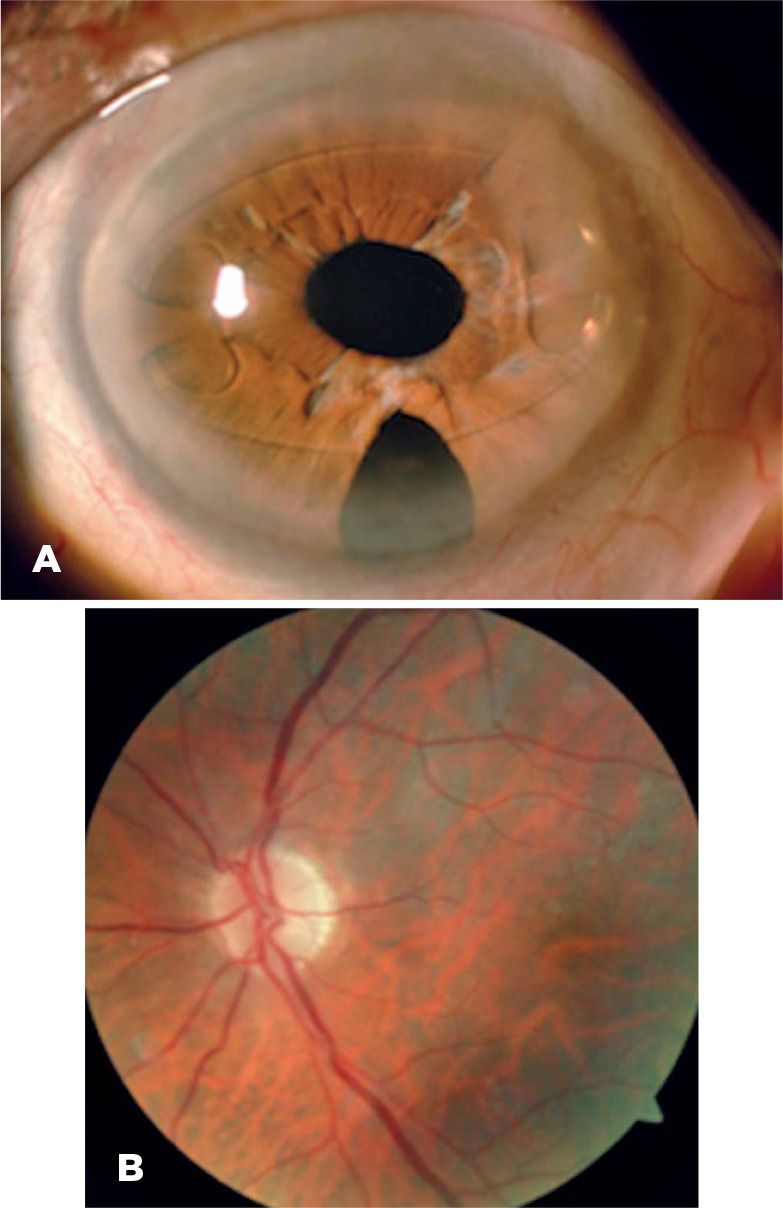
The only patient with favorable results in the series (case 6): A clear cornea, an iris-fixated intraocular lens, and a large peripheral iridectomy were noted on the slit-lamp photograph (A), whereas paleness of the optic disc was visible on color fundus image at 10-year follow-up (B).

The only patient who had a good functional outcome did not receive keratoplasty and had no systemic or ocular disease (Figure 1). In our series, this patient had the fewest surgical interventions (Case 6). Typically, a worse long-term outcome was associated with a greater number of surgical interventions (Cases 3 and 4).

In the eyes in which fundoscopy could be performed during the last examination, paleness of the optic disc was observed to varying degrees. In those patients, advanced age, accompanying systemic diseases, presumed toxicity of the intravitreally administered antifungal drugs, injury from multiple surgeries, and extensive inflammatory reaction in the acute phase all might have contributed to the development of optic neuropathy.

In conclusion, in the treatment of fungal endophthalmitis, successful eradication in the early period was achieved using intensive antifungal therapy and multiple surgical interventions. Despite the additional surgeries performed for visual rehabilitation in our series, only one affected eye maintained useful vision in the long-term.
